# *Mycobacterium tuberculosis* and *M. bovis* BCG Moreau Fumarate Reductase Operons Produce Different Polypeptides That May Be Related to Non-canonical Functions

**DOI:** 10.3389/fmicb.2020.624121

**Published:** 2021-01-12

**Authors:** Marcos Gustavo Araujo Schwarz, Deborah Antunes, Paloma Rezende Corrêa, Antônio José da Silva-Gonçalves, Wladimir Malaga, Ernesto Raul Caffarena, Christophe Guilhot, Leila Mendonça-Lima

**Affiliations:** ^1^Laboratório de Genômica Funcional e Bioinformática, Instituto Oswaldo Cruz, Fiocruz, Rio de Janeiro, Brazil; ^2^Laboratório Interdisciplinar de Pesquisas Médicas, Instituto Oswaldo Cruz, Fiocruz, Rio de Janeiro, Brazil; ^3^Institut de Pharmacologie et de Biologie Structurale, Université de Toulouse, CNRS, Université Paul Sabatier, Toulouse, France; ^4^Grupo de Biofísica Computacional e Modelagem Molecular, Programa de Computação Científica, Fiocruz, Rio de Janeiro, Brazil

**Keywords:** fumarate reductase, *Mycobacterium bovis* BCG Moreau, homopolymeric sequence, transcriptional slippage, tuberculosis vaccine

## Abstract

Tuberculosis is a world widespread disease, caused by *Mycobacterium tuberculosis* (*M.tb*). Although considered an obligate aerobe, this organism can resist life-limiting conditions such as microaerophily mainly due to its set of enzymes responsible for energy production and coenzyme restoration under these conditions. One of these enzymes is fumarate reductase, an heterotetrameric complex composed of a catalytic (FrdA), an iron-sulfur cluster (FrdB) and two transmembrane (FrdC and FrdD) subunits involved in anaerobic respiration and important for the maintenance of membrane potential. In this work, aiming to further characterize this enzyme function in mycobacteria, we analyzed the expression of FrdB-containing proteins in *M.tb* and *Mycobacterium bovis* Bacillus Calmette–Guérin (BCG) Moreau, the Brazilian vaccine strain against tuberculosis. We identified three isoforms in both mycobacteria, two of them corresponding to the predicted encoded polypeptides of *M.tb* (27 kDa) and BCG Moreau (40 kDa) *frd* sequences, as due to an insertion on the latter’s operon a fused FrdBC protein is expected. The third 52 kDa band can be explained by a transcriptional slippage event, typically occurring when mutation arises in a repetitive region within a coding sequence, thought to reduce its impact allowing the production of both native and variant forms. Comparative modeling of the *M.tb* and BCG Moreau predicted protein complexes allowed the detection of subtle overall differences, showing a high degree of structure and maybe functional resemblance among them. Axenic growth and macrophage infection assays show that the *frd* locus is important for proper bacterial development in both scenarios, and that both *M.tb*’s and BCG Moreau’s alleles can partially revert the hampered phenotype of the knockout strain. Altogether, our results show that the *frdABCD* operon of Mycobacteria may have evolved to possess other yet non-described functions, such as those necessary during aerobic logarithmic growth and early stage steps of infection.

## Introduction

Tuberculosis (TB) remains as an important health issue and is among the 10 diseases in death numbers worldwide. Despite the availability of early diagnostic tools and well-established treatment protocols, TB remains a global health emergency, as declared in 1993 by the World Health Organization (WHO) (Dolin et al., 1993). Since 2015, this scenario has aggravated and TB surpassed the human acquired immunodeficiency syndrome, reaching the first place in death numbers caused by an infectious contagious disease (World Health Organization, 2019). Its causative agent is *Mycobacterium tuberculosis* (*M.tb*), able to cause lesions mainly in host lungs but also in other tissues during active disease when it is in replicative state. In the early infection phase, bacteria are taken-up by macrophages and other immune cells, where they encounter life-limiting conditions such as low oxygen tension and nutrient availability that can lead *M.tb* to a non-replicative state (Salgame et al., 2015). It is known that *M.tb* displays a high capacity to adapt to these different environments, a feature also represented by its repertoire of enzymes involved in energy metabolism.

As an obligate aerobe, it is expected that *M.tb* would use respiration (electron transport chain and oxidative phosphorylation) as the primary source for ATP production and regeneration of reducing equivalents, where the exposure to long-term microaerophilic situations would lead to metabolically inactive bacteria. However, we know that the bacteria residing inside granulomas can restore active disease, thus re-entering a replicative state when proper conditions are met (Cadena et al., 2017). So, *M.tb*, as other mycobacteria, possesses a range of terminal reductases to maintain redox homeostasis while a set of dehydrogenases are fueling different oxidative energy-producing metabolisms in oxygen-limiting conditions. One of these final reductases predicted to be involved in anaerobic respiration is fumarate reductase (FRD), a fumarate-reducing enzyme that restores some reducing equivalents (Cook et al., 2014).

**FIGURE 1:**
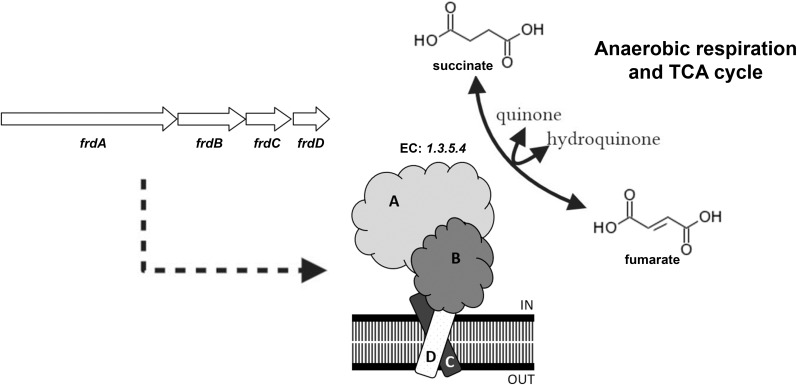
Scheme of fumarate reductase, showing operon structure in *M.tb* and predicted complex organization in the plasma membrane. This enzyme (EC: 1.3.5.4) catalyzes the redox interconversion of succinate to fumarate, using quinones as secondary substrate. This enzymatic activity is important to anaerobic respiration in some prokaryotes, as well as to the tricarboxylic acid (TCA) cycle. Reaction scheme adapted from Kyoto Encyclopedia of Genes and Genomes (KEGG).

Fumarate reductase is structurally and mechanistically similar to succinate dehydrogenase (SDH), the respiratory chain complex II (Maklashina and Cecchini, 2010). These enzymes catalyze the redox interconversion of succinate and fumarate, requiring several coenzymes, such as flavin adenine dinucleotide (FAD) and quinones, and prosthetic groups. For some organisms having both complexes, each one is used in a specific reaction direction and under specific conditions (Cecchini et al., 2002). Besides its role in redox maintenance (Rao et al., 2008), FRD is thought to be important to generate succinate that could be secreted in order to maintain membrane potential in oxygen-limiting, non-replicative Mycobacteria (Watanabe et al., 2011; Figure 1).

The only available prophylactic measure against tuberculosis is vaccination with Bacillus Calmette–Guérin (BCG), a *Mycobacterium bovis* attenuated strain. Due to several reasons, a genetically diverse group of BCG strains is used today for vaccine production worldwide, with different vaccine properties (Abdallah et al., 2015). The strain used in Brazil from 1928 until 2017 is *M. bovis* BCG Moreau, phylogenetically close to BCG Russia and Japan, all of them considered to be more immunogenic and closer to the original BCG. Our group has been working on its molecular characterization, also using it as a model to better understand mycobacterial biology (Berrêdo-Pinho et al., 2011; Gomes et al., 2011; Galvão et al., 2014; Monteiro-Maia et al., 2018; Pagani et al., 2019). Analysis of the FRD operon DNA sequence in BCG Moreau shows a mutation in a poly-G homopolymeric region at the end of *frdB*, when compared to *M.tb*. This leads to a sequence coding for a hypothetical fused FrdBC protein in several members of the *M. bovis* lineage, not yet functionally analyzed. Here we report the characterization of BCG Moreau FRD, comparing to *M.tb*. Additionally, FRD function was assessed using a Δ*frd* knockout strain (Schwarz et al., 2020) and complementing it with the alleles from both BCG Moreau and *M.tb*, verifying differential phenotypes under laboratory growth conditions and in macrophage infection assays, such as a delay in axenic growth and a lower internalization rate for the knockout strain. In parallel, structural comparison of both FRD complexes shows subtle overall differences, indicating a theoretically high function similarity. Altogether, the data presented here contribute to the characterization of FRD and its function(s), adding to the discussion on the impact of mutations on bacterial genes, mainly focusing on poly-G/C regions inside coding sequences.

## Materials and Methods

### Sequence Analysis

*Mycobacterium tuberculosis* H37Rv *frdB* and *frdC* region sequences (accession NC_000962.3, coordinates 1759435.1760555) were obtained from the public domain databank NCBI. The *M. bovis* BCG Moreau corresponding sequence was found by Blast analysis, identifying BCG_M1572/FrdBC (coordinates 1744745.1745875) on the *M. bovis* BCG str. Moreau RDJ complete genome (GenBank: AM412059.2). Nucleic acid and protein alignments were performed with the free online tool Clustal Omega.

### Recombinant Protein Expression, Purification and Polyclonal Sera Production

For recombinant FrdB production, the sequence corresponding to *M.tb*’s *frdB* (position 1744752.1745470 on BCG Moreau genome) was cloned in pET28a(+), adding a C-terminal 6-His tag to the final recombinant protein. *M. bovis* BCG Moreau genomic DNA was used as template to amplify a fragment containing *Nco*I and *Xho*I restriction sites, using Platinum SuperFi DNA polymerase (Invitrogen) and the following pair of primers: AAA**CCATGG**ATCGAATTGTCATGG (forward) and TTT**CTCGAG**GAACAGCAACTTCTT (reverse).

After selection of the desired DNA construct on *Escherichia coli* TOP10 and sequence confirmation, plasmid DNA was transformed into *E. coli* BL21(DE3) for recombinant protein expression. Cells were harvested after induction with 1 mM isopropyl-β-D-thiogalactopyranoside (IPTG) for 3 h at 37°C, on Luria Bertani (LB) medium containing 25 μg/mL kanamycin.

His-tagged protein was purified by Immobilized Metal Affinity Chromatography (IMAC) on a HisTrap HP 1 mL column (GE Healthcare), charged with Ni^2+^. Elution was performed by 5 column volumes (CV) step-gradient (10, 20, 30, 40, and 100%) with elution buffer (same as loading, but with 500 mM imidazole). Fractions were analyzed by 12% SDS-PAGE and those containing purified protein were pooled, dialyzed against PBS and used to immunize six BALB-C mice, essentially as described (Castro-Pinto et al., 2008). Whole individual mouse serum was collected and tested against purified recombinant BCG His-tagged protein on western blotting assays to confirm antibody production. Responding sera were pooled and used as FrdB polyclonal antibody (α-FrdB). This study was approved by the Commission for the Use of Laboratory Animals from IOC, Fiocruz (License CEUA/IOC L-020/2016).

### Native Frd In-Gel Characterization

*Mycobacterium tuberculosis* H37Rv and *M. bovis* BCG Moreau cells were grown on 7H9 medium supplemented with 10% ADC (albumin, dextrose, and catalase) at 37°C under agitation (200 rpm). Cultures were started at O.D._600 *nm*_∼0.1 and cells from samples at lag, logarithmic and stationary growth phases recovered by centrifugation. Cells were resuspended in lysis buffer (50 mM HEPES/KOH pH 7.5; 10 mM MgCl_2_; 60 mM NH_4_Cl; 10% [v/v] glycerol) and submitted to 3 lysis cycles (1 min each) on a Bead Beater apparatus (Bio101), using glass beads. The clarified lysate (total protein fraction) was obtained after centrifugation (15,000 × *g* for 10 min). Total soluble proteins were quantified using NanoDrop and 20 μg resolved by 15% SDS-PAGE prior to western blotting with α-FrdB polyclonal antibody. Briefly, proteins were transferred to nitrocellulose membrane (Hybond C, GE) using a Mini Trans-blot equipment (Bio-Rad) at constant 100 V for 1 h. The quality of protein transfer was assessed by reversible staining with Mem Code (Pierce) and the membrane blocked for 16 h at 4°C in TBS-T (Tris-buffered saline supplemented with 0.05% (v/v) Tween 20) containing 10% (w/v) skim milk. After washing with TBS-T, the membrane was incubated with α-FrdB (1:1,000) for 2 h, followed by incubation with HRP-conjugated goat anti-mouse IgG (1:10,000) for 1 h. After each antibody incubation, membrane was washed three times with TBS-T, followed by three washes with TBS. All antibody incubations were performed in a 5% (w/v) skim milk solution in TBS-T. Blots were developed using SuperSignal kit (Pierce), following manufacturer protocol.

For thermostability assays, total proteins from logarithmic phase cell lysates were incubated for 30 min at 40, 80, or 100°C, centrifuged at 15,000 × *g* for 10 min, and protein content from supernatant was quantified and used as sample for western blotting. We also performed differential extraction with Triton X-114 [as described on Målen et al. (2010)] with BCG Moreau samples from logarithmic growth phase. Briefly, Triton X-114 was added to clarified lysates (final concentration of 2% [v/v]) and incubated under agitation at 4°C for 16 h. After this, sample was incubated at 37°C for 40 min and phase separation was performed by centrifugation at 1,000 × *g* for 20 min. After protein precipitation, pellets from aqueous and detergent phases were dissolved in 8 M urea and 2% (v/v) CHAPS prior to quantification and western blotting.

### Complementation of the *M. bovis BCG* Moreau *frdABCD* Knockout and Growth in Axenic Medium

We performed a homology recombination-based recombineering assay to create the *frdABCD* knockout strain of *M. bovis* BCG Moreau, as described in Schwarz et al. (2020). Briefly, the allelic exchange substrate (AES) (1 kb of up- and down-stream regions adjacent to *frd* operon flanking the *kanR* gene) was transformed on electrocompetent *M. bovis* BCG Moreau cells harboring pJV53H after induction with acetamide. Recombination events were selected after plating on 7H11 plates supplemented with 10% OADC (oleic acid, albumin, dextrose, and catalase) and kanamycin (40 μg/mL). Locus structure was tested by PCR with primers annealing inside the excised region and inside *kanR* and the 1 kb up- and down-stream sequences.

Complementation with BCG Moreau and *M.tb frdABCD* alleles and corresponding promoters was achieved after cloning *Spe*I-digested fragments containing the open reading frames (ORF) plus 1 kb upstream (1741928.1746299 for BCG Moreau and 1756622.1760984 for *M.tb* H37Rv) on the integrative mycobacterial vector pMV361H (Stover et al., 1991). Inserts were sequenced by Sanger technology to confirm each allele structure and plasmid DNA transformed on electrocompetent *M. bovis* BCG Moreau Δ*frd* cells and selected on kanamycin/hygromycin (50 μg/mL) plates.

For axenic growth analysis, wild-type (WT), *frdABCD* knockout (Δ*frd*) and Δ*frd* complemented with the alleles from *M.tb* and BCG Moreau (Δ*frd*:*frdM.tb* and Δ*frd*:*frd*BCGM, respectively) strains were grown on 7H9 medium supplemented with 10% ADC at 37°C. Cultures were launched with an O.D._600 *nm*_ of 0.1, and turbidometry analysis was performed. To assess growth velocity within logarithmic growth phase we performed a linear regression analysis with these points and calculated and compared curve slopes. We also compared the curve-intercepting *x*-axis values, indicative of entry into logarithmic growth phase.

For western blotting assays, stationary phase cells were processed as described in section “Native Frd In-Gel Characterization.” Polyclonal serum against Rv1373 (1:1,000) was used as a positive loading control.

### Macrophage Infection Assays

J774 murine macrophages were grown on RPMI 1640 medium supplemented with 10% fetal bovine serum and 1% of amino acid solution 50× (Sigma Aldrich). For the infection assays, 2 × 10^5^ cells were incubated with bacterial strains (M.O.I. 10:1) for 4 h at 37°C and 5% CO_2_. Non-internalized bacteria were removed by three washes with medium and the cells were incubated as described. Bacterial samples were recovered at different time points (4, 6, 24, 48, 72, and 96 h) by differential lysis of macrophages with 0.05% SDS solution, and after clarification by centrifugation serial dilutions (−2 and −3) were plated on 7H10 medium supplemented with 10% ADC for CFU counting after 3 weeks incubation at 37°C.

### Statistical Analysis

Results are represented as the mean of a set of three independent experiments, with standard deviation, and for statistical analysis, we performed a one-way or two-way ANOVA on GraphPad Prism 5 software.

### Comparative Modeling and Structural Analysis

The BLASTP program (Altschul, 1997) was applied to select FRD structures to be used as templates available in the Protein Data Bank (PDB) for comparative modeling. The Quinol-FRD of *E. coli* (Benkert et al., 2009; Bond and Schüttelkopf, 2009) in complex with inhibitor 2-heptyl-4-hydroxy quinoline N-oxide (HQO), FAD, oxaloacetate (OAA) and iron/sulfur clusters [PDB ID: 1KF6 (Iverson et al., 2002)] was selected as a template for FRD targets.

Target and template sequences were then aligned using the PSI-Coffee mode using the T-Coffee program (di Tommaso et al., 2011). For each target sequence, five hundred homology models were created using the standard “*auto model*” routine of the Modeler program version 9.20 (Webb and Sali, 2014), with heteroatoms. Each model was optimized via the variable target function method (VTFM) until 300 iterations were achieved. Molecular dynamics optimization was conducted in the *slow-level* mode.

After repeating the full cycle twice, the resulting modeled structures were selected according to their discrete optimized protein energy (DOPE) score.

Models were evaluated by the QMean server (Benkert et al., 2009), while the ERRAT graphics and Ramachandran plots were calculated using the SAVES server of UCLA-DOE Lab^1^ for stereochemical analysis. The TM-align algorithm was used for checking structural alignment and comparing models. The protein-ligand 2D interactions maps were analyzed using the PoseView (Stierand and Rarey, 2010) tool of the Proteins Plus server (Fährrolfes et al., 2017)^2^. The sequence alignment figure was generated through ALINE (Bond and Schüttelkopf, 2009) software. The orientation of FRD in the membrane was predicted using the *PPM server* in the *OPM* database server (Lomize et al., 2012)^3^. Figures corresponding to the three-dimensional structures were generated using UCSF ChimeraX software (Goddard et al., 2018).

## Results

### A 10 bp Poly-G Insertion in BCG Moreau Leads to the Fusion of FrdB and FrdC Subunits

**FIGURE 2:**
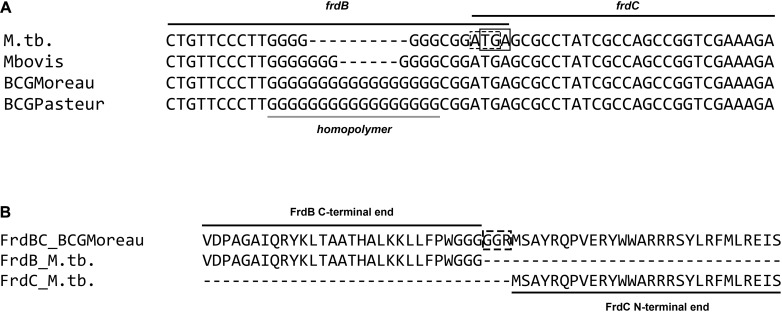
Alignment of the *frd* poly-G homopolymer region. **(A)** DNA sequence alignment of the *frdB*-*frdC* region of *M.tb*, *M. bovis* and *M. bovis* BCG strains Moreau and Pasteur, showing a variable poly-G homopolymer at the 3′ end of *frdB*, leading to the fusion of Frd subunits B and C in BCG Moreau **(B)**.

DNA sequence alignment of the *M.tb frdB* and *frdC* regions (*rv1553_rv1554*–1760155. 1760204 on the NC_000962.3 *M.tb* H37Rv complete genome) to BCG Moreau (BCG_M1572) and Pasteur (BCG_1605) shows a 10 bp poly-G insertion in BCG. This insertion occurs in an already G-rich sequence at the end of the *frdB* ORF and, as shown in Figure 2A, leads to the loss of the stop codon for *frdB* and to an in-frame fusion with FrdC. Both regions (*M.tb* and BCG Moreau alleles) were re-sequenced prior to further assays to confirm each pattern. Genome annotation predicts a merged FrdBC protein in BCG Moreau with 100% N-terminal and C-terminal sequence homology to *M.tb*’s FrdB and FrdC, respectively (Figure 2B).

**FIGURE 3:**
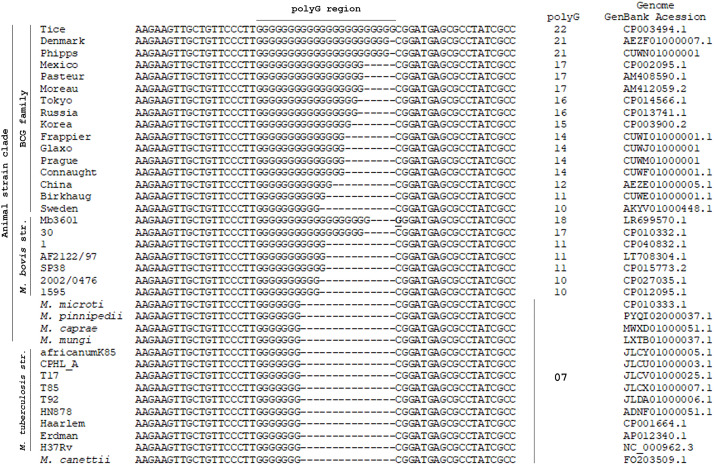
Alignment of the *frd* poly-G homopolymer region from several mycobacterial deposited genomes throughout the *Mycobacterium tuberculosis* complex. As can be observed, high variability and longer G stretches (from 10 to 22 G nucleotides) can be detected on the *M. bovis* lineage, including the BCG family. On the remaining clades within the complex the *M.tb* H37Rv pattern (7 G nucleotides) is observed. The GenBank Accession number is provided for each genome analyzed.

Comparison to other closely related mycobacteria shows the same insertion pattern but with different size in the case of *M. bovis* AF2122/97 (Mb1579) (Figure 2A). Analysis of other species belonging to the *M. tuberculosis* complex shows that this mutation is only present in the *M. bovis* lineage, and the *M.tb* H37Rv pattern is observed throughout *M.tb* lineages 1 to 4, the *Mycobacterium africanum* 1 and 2 lineages, and the remaining species of the animal strain clade on the *M. tuberculosis* complex phylogeny (Figure 3). In contrast, sequence variability within this poly-G region is found throughout other publicly available *M. bovis* and BCG strain genomes.

### The *frd* Operon Produces Three FrdB-Containing Isoforms With Different Physico-Chemical Characteristics

**FIGURE 4:**
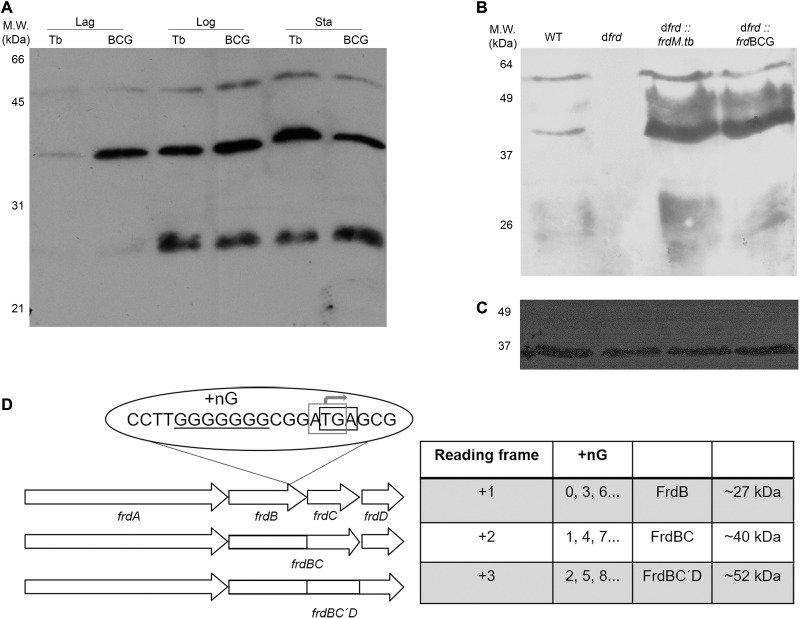
Physico-chemical characterization of FrdB-containing isoforms in *M.tb* (Tb) and BCG Moreau (BCG) protein extracts. Prior to western blotting with α-FrdB, 20 μg of proteins from each sample were resolved on 15% SDS-PAGE. **(A)** Expression during axenic growth was analyzed on total protein extracts obtained from Lag, logarithmic (Log) and stationary (Sta) phases. Western blotting assays with α-FrdB **(B)** and α-Rv1373 (**C**; loading control), showing that under our assay conditions the BCG Moreau *frd* knockout (d*frd*) has no FrdB-reactive isoforms, while complemented strains with *M.tb* (d*frd*:*frdM.tb*) and BCG Moreau (d*frd*:*frd*BCG) alleles reacquire the wild-type (WT) three band pattern. **(D)** Scheme explaining the RNA polymerase slippage scenario. Three possible reading frames arise depending on the number of G nucleotides (+nG), generating fusion products (FrdBC in frame +2 or FrdBCD in +3, but with *frdC* out-of-frame), or each protein expressed separately (frame +1).

As can be observed in Figure 4A, western blotting with α-FrdB detects three FrdB-containing isoforms (27, 40, and 52 kDa), both in *M.tb* and BCG Moreau, mainly in the logarithmic and stationary growth phases. The 27 and 40 kDa bands should correspond to FrdB and to the fused FrdBC proteins, predicted for *M.tb* and BCG Moreau, respectively. The third, 52 kDa FrdB-reactive band coincides with the expected MW for a FrdBCD fused polypeptide (Figure 4D). Expression of the three FrdB- isoforms is abolished in a BCG Moreau *frd* knockout strain (Δ*frd*), under our experimental conditions, a phenotype readily restored to wild-type upon complementation with either the *M.tb* or BCG Moreau *frd* alleles (Figures 4B,C).

**FIGURE 5:**
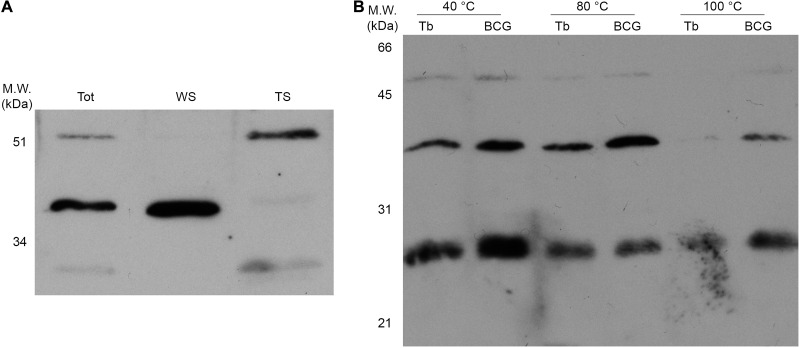
Physico-chemical characterization of the three FrdB-containing isoforms in protein extracts. Prior to western blotting with α-FrdB, 20 μg of proteins from each sample were resolved on 15% SDS-PAGE. **(A)** Protein samples from BCG Moreau Log phase (Tot) were used to further characterize differential partition of isoforms on water-soluble (WS) or Triton-soluble (TS) fractions after Triton X-114 extraction. **(B)** Thermostability assay were performed by incubating proteins extracts at 40, 80, and 100°C for 30 min, centrifugation and fractionation of soluble proteins by SDS-PAGE prior to western blotting.

Differential extraction with Triton X-114 leads to the detection of the majority of the 52 and 27 kDa α-FrdB-reactive bands in the organic phase (Figure 5A), indicative of their higher hydrophobicity. Thermostability in-gel assay (Figure 5B) also shows a differential pattern for these isoforms, with higher molecular weight proteins being less thermostable under the tested conditions.

### Structural Models of *M.tb* and BCG Moreau FRD Complexes Show Subtle Overall Differences

We have built three-dimensional models of the FRD complex from *M.tb* (with FrdB) and BCG Moreau (with FrdBC) based on the Quinol-FRD of *E. coli* crystallographic protein structure, to evaluate the influence of the GGR link connecting subunits B and C on the BCG Moreau structure. Analysis via BLASTP (Supplementary Table 1), concerning identity, similarity, coverage, gaps, max score and *e*-values for each chain for *M.tb* FRD, showed higher conservation for chains A and B (with 55 and 50% identity, respectively) and lesser conservations for chains C and D (with 31 and 40% identity, respectively). The multiple sequence alignment between FRD target sequences and PDB template sequence is shown in Supplementary Figure 1. The quality of the structural models was assessed according to the QMean, ERRAT2 and Ramachandran plot evaluations (Supplementary Table 2).

**FIGURE 6:**
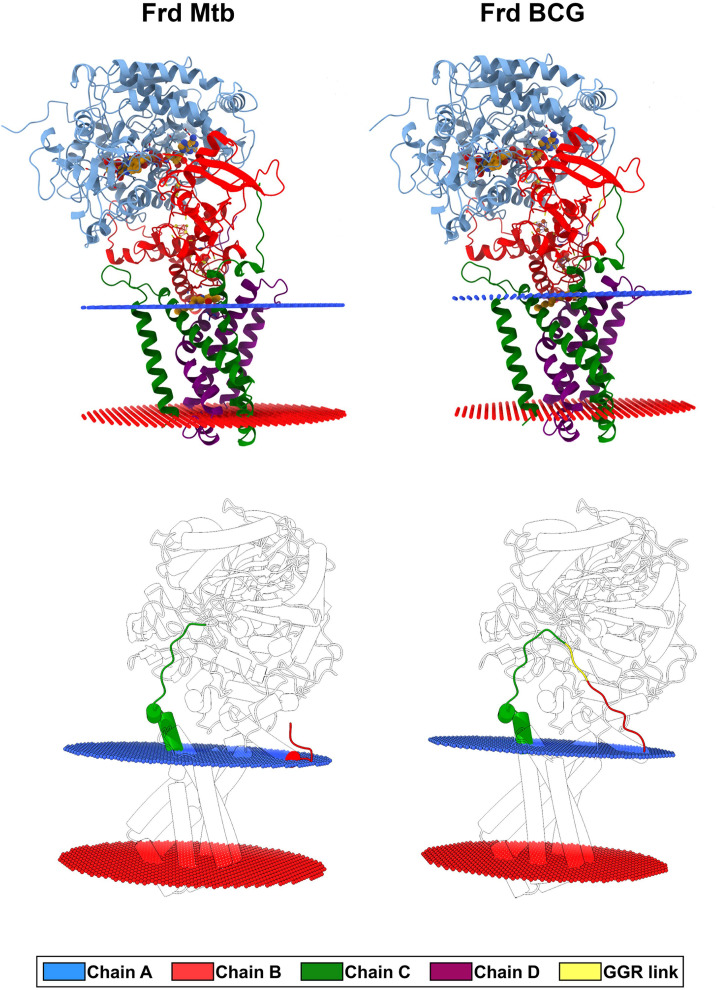
Three-dimensional models of the FRD complex obtained by comparative modeling (top panel), and spatial location of the GGR link connecting the predicted fused FrdB and FrdC parts on the BCG Moreau FRD structure (bottom panel). Red and blue rings show the position of the lipid/head-group region of the periplasmic and cytoplasmic leaflets of the membrane bilayer, respectively. Chains and GGR link are highlighted in cartoon representation and colored according to the figure legend caption.

Results showed that *M.tb* and BCG Moreau FRD structures present subtle overall differences (Figure 6, top panel), according to the low root-mean-square deviation (RMSD) value (0.88 Å) and high TM-score (0.96), meaning that both structures share a characteristic folding and high conservation. The RMSD is a measure of the structural distance between two superimposed three-dimensional structures where an RMSD = 0.0 indicates a perfect match between two structures as a TM-score of 1 also does.

**FIGURE 7:**
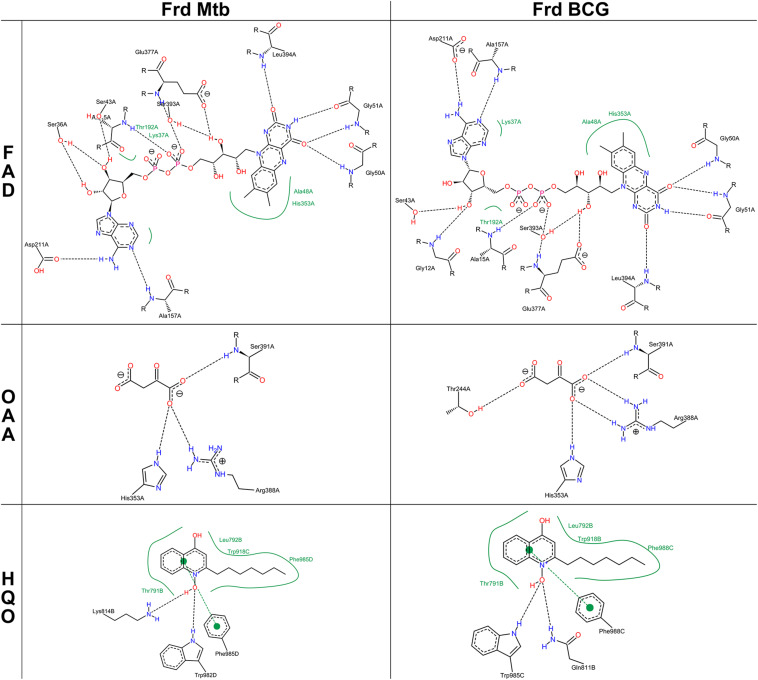
2D-interaction maps of FRD models with flavin-adenine dinucleotide (FAD), oxaloacetate (OAA), and 2-heptyl-4-hydroxy quinoline N-oxide (HQO) were generated using PoseView server. Black dashed lines and full green lines show hydrogen bonds and hydrophobic interactions, respectively.

The most prominent alteration of the BCG Moreau structure when compared to *M.tb*’s resides in the GGR link connecting FrdB and FrdC parts (Figure 6, bottom panel), altering the protein orientation in the membrane having a higher hydrophobic thickness (31.2 ± 1.2 Å) and a tilt angle (6 ± 1°) in relation to the *M.tb* FRD structure (28.8 ± 1.9 Å and 5 ± 1°) (Figure 6). Locally, however, this extra protein segment does not cause a significant impact on the 3D structure of the complex. When looking particularly to amino acids interacting with known coenzymes and inhibitors of FRD (such as FAD, OAA, and HQO), we observe local changes in the intermolecular interactions, that are all related to rotations in the residues side chain (Figure 7).

### The *frd* Operon Is Important for Proper Growth on Axenic Medium and During Macrophage Infection

**FIGURE 8:**
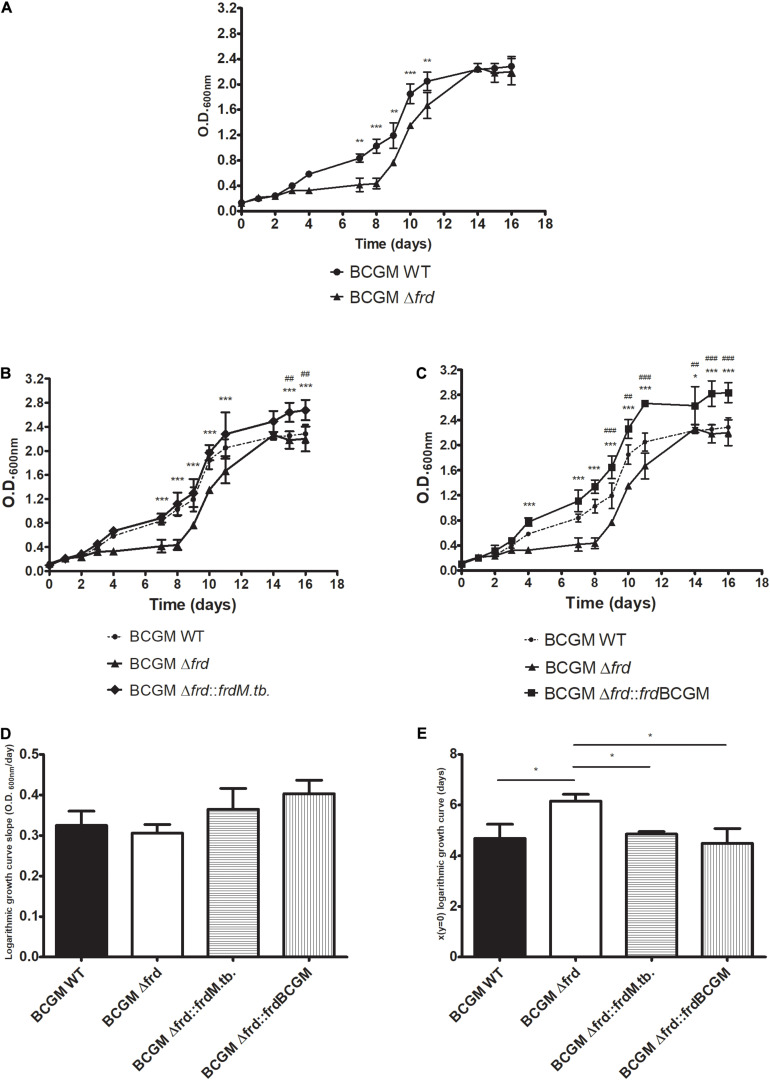
Axenic growth profile of wild-type (WT), *frd* knockout (Δ*frd*) and complemented (Δ*frd*:*frdM.tb* and Δ*frd*:*frd*BCGM) strains on 7H9 medium. Effect of operon excision **(A)** and complementation with the alleles from *M.tb* **(B)** and BCG Moreau **(C)** on growth characteristics. **(D)** Analysis of growth velocity (curve slope) on logarithmic phase shows no difference among strains, **(E)** but entrance into this growth phase seems to be delayed on Δ*frd* strain. In panels **(B,C)**, significant differences between complemented and wild type (#) or knockout (*) strains are indicated. *P*-values: (*) < 0.05; (**, ##) < 0.01; (***, ###) < 0.001. Data represent the mean ± SD of three independent experiments.

In order to better understand the impact of the *frd* operon, a BCG Moreau knockout previously constructed (Δ*frd*) (Schwarz et al., 2020) by removal of genes *frdABCD*, was used. The knockout strain was complemented with the BCG Moreau (Δ*frd*:*frd*BCGM) or *M.tb* (Δ*frd*:*frdM.tb*) *frdABCD* alleles, including 1 kb of upstream region, with 99% sequence identity. Analysis of the axenic growth profile from these strains indicates a clear difference between the knockout and WT strains, with Δ*frd* showing significant lower absorbance levels during logarithmic growth phase (days 7–11), but with similar values when stationary phase is reached (days 14–16) (Figure 8A). This log-phase related phenotype is restored to WT levels when complementing Δ*frd* with both the *M.tb* and BCG Moreau *frd* alleles, with higher absorbance levels for the complemented strains when compared to WT in final axenic growth phases (Figures 8B,C).

Comparison of the slopes from the logarithmic growth phase curves shows no significant differences (Figure 8D), but significant higher values are observed when analyzing the curve-intercepting *x*-axis values for the Δ*frd* strain (Figure 8E), indicating a delay in entering logarithmic growth for the knockout strain.

**FIGURE 9:**
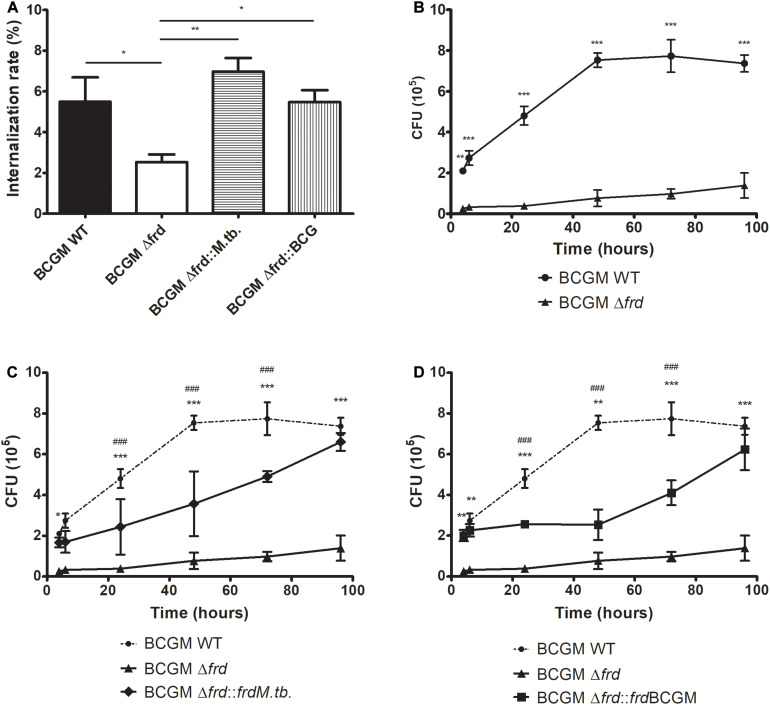
Impact of *frd* operon loss on BCG internalization **(A)** and survival in a macrophage model of infection. Wild-type (WT), *frd* knockout (Δ*frd*) and complemented (Δ*frd*:*frdM.tb* and Δ*frd*:*frd*BCGM) BCG Moreau strains were used to infect murine J774 macrophages. An impairment on growth is detected in Δ*frd* **(B)**, and phenotype is partially restored to WT levels by complementation of knockout strain with both *M.tb* **(C)** and BCG Moreau **(D)** alleles. Internalization rate is expressed as the number of bacteria associated with J774 cells at 4 h p.i./number of bacteria in the initial inoculum. In panels **(C,D)**, significant differences between complemented and wild type (#) or knockout (*) strains are indicated. *P*-values: (*) < 0.05; (**) < 0.01; (***, ###) < 0.001. Data represent the mean ± SD of three independent experiments.

Due to the growth delay phenotype observed for the knockout strain in axenic medium, we further verified if this deletion could lead to a differential feature in a macrophage infection scenario. As shown in Figure 9, the Δ*frd* knockout strain shows a significant decrease in macrophage internalization rate values when compared to the other strains (Figure 9A). Furthermore, deletion of the *frd* operon leads to lower bacterial survival after infection, at all time points analyzed, when compared to WT (Figure 9B), and this phenotype is partially, but not fully reversed when complementing with both alleles tested (Figures 9C,D).

## Discussion

Fumarate reductase is considered an important enzyme regarding anaerobic respiration, and therefore thought to be necessary, on the mycobacterial context, for successful development of the microorganism during infection of the host cell, where the bacteria face a microaerophilic environment. DNA sequence analysis of the BCG Moreau *frdABCD* operon shows a 10 bp insertion in a G-rich region at the end of *frdB* resulting in a predicted fused FrdBC protein product, when compared to *M.tb* H37Rv. This led us to investigate the possible functional impacts of this insertion onto the biology of the Brazilian vaccine strain.

Comparison to DNA sequences from other related mycobacteria indicates that mutations on this locus occur only in the *M. bovis* lineage (and therefore, in all BCG strains), but with variable numbers of repetitive nucleotides within the insertion, from 10 to 22 Gs. It is important to keep in mind that, due to its homopolymeric composition, this G-stretch is prone to sequencing errors, and the exact number of repetitive nucleotides within it may not be accurate in all genomes analyzed. It is already known that repetitive sequences are prone to mutations, mainly when considering insertions/deletions (INDELs), and this is thought to occur due to DNA polymerase slippage. As described for *M.tb* (Dechering, 1998), despite being a high G/C content bacterium, poly-G or -C regions are underrepresented in its genome, possibly indicating that replication slippage would work less efficiently on expanding repetitive nucleotides on G/C-rich sequences. This is believed to result from the more stable interaction between G/C pairs than A/T, since slippage requires local melting of the DNA duplex, favoring events on polyA/T sequences. Nonetheless, we found a polyG region that seems to be prone to replication slippage, as reflected by the diversity in the number of repetitive nucleotides within this sequence in different members of the *M. bovis* lineage.

Analysis of the western blot results identified FrdB-reactive bands, mainly on logarithmic and stationary growth phases, corresponding to FrdB (∼27 kDa), FrdBC (∼40 kDa) and a higher band of ∼52 kDa, on protein samples from both *M.tb* and BCG. Western blotting analysis with the *frd* knockout and complemented strains reinforces that these three FrdB-containing bands are indeed produced by the *frd* operon, as the Δ*frd* protein extract is not reactive when using FrdB polyclonal serum, while wild-type and complemented strains are. This three-band pattern raises the hypothesis of polymerase slippage at the transcriptional level, in which both alleles generate a pool of mRNAs with different nucleotide numbers on the G-rich region. If such is the case, all transcripts would produce a protein sequence containing FrdB (either as a single polypeptide or as the N-terminal part of one). After the G-stretch, three different scenarios would be possible, corresponding to three different reading frames (Figure 4D). Frame 1 corresponds to the *M.tb* sequence, leading to the production of FrdB, FrdC, and FrdD as separated subunits. Frame 2 would lead to a fused FrdBC product and a separate FrdD subunit. Finally, frame 3 would yield a ∼52 kDa protein, corresponding to what we are calling FrdBC’D, with an N-terminal part identical to FrdB fused to an intermediate “out-of-frame” protein sequence with no homology to known proteins (FrdC’), followed by a C-terminal part identical to the FrdD subunit, since *frdB* and *frdD* would retain the original reading frames.

This hypothesis is corroborated by already published works on transcriptional slippage (Baranov et al., 2005), in which slippage-prone sequences in coding regions lead to a pool of mRNAs that can produce different proteins after the repetitive region. This is believed to be a deleterious event and one of the causes of several known disorders, as is the case of the production of aberrant isoforms of beta-amyloid protein in Alzheimer’s syndrome patients (van Leeuwen et al., 1998). There are two predicted scenarios in which slippage could be advantageous. The first one is when the different protein products have different and useful functions, meaning that from a single gene more than one polypeptide can be produced, as is the case for some Ebola and Paramyxovirus coding regions (Hausmann et al., 1999; Volchkov, 2001). The second is when the slippage event restores a reading frame disrupted by an upstream mutation (Wons et al., 2015). Rockah-Shmuel et al. (2013) observed a correlation between the occurrence of INDELs in coding sequences and a higher frequency of transcriptional slippage events within these sequences, believed to be advantageous since it would restore the original protein structure and activity, disrupted by the INDEL, by producing it among other variants (Rockah-Shmuel et al., 2013). Analyzing our data, it is possible to speculate that the production of three FrdB-containing proteins is an ancient phenotype within Mycobacteria, as *M.tb*, with the 7 G’s genotype, generates the same protein pattern as BCG Moreau, with a 17 G’s genotype. If this is so, variability within this stretch found in the *M. bovis* lineage does not seem to result from natural selection.

We also developed structural models for FRD complexes with FrdB and FrdC as separated proteins (the “*M.tb* case”) or fused (the “BCG scenario”). We could not create models for FrdBC’D as there is yet no available homologous structure for the out-of-frame *frdC*-coded region for comparative modeling. Analysis of both structures shows no significant differences between them, as to coenzymes and inhibitor (FAD, OAA, and HQO) interacting-amino acids, although local changes in the intermolecular interactions could be detected. Further analyses are needed to verify the possible consequences of these small changes on the regulation of FRD activity. Nonetheless, this structural resemblance could be related to the fact that both microorganisms produce both isoforms, meaning that they can still be similarly functional and producing FrdBC would not be deleterious for the bacteria.

The three FrdB-containing isoforms show clear differences as to their physical-chemical characteristics. FrdBC’D partitions to the organic phase after differential extraction with Triton X-114, an indication that it is more hydrophobic than the other isoforms. This could be due to the presence of FrdD, the other FRD complex membrane anchor, or to its higher molecular weight. Since it is the only isoform for which we do not have any evidence of structure/activity, the possibility that it remains highly insoluble due to random structures and exposure of hydrophobic regions cannot be ruled out. Thermostability, on the other hand, decreases as the molecular weight of the different isoforms increases.

In an effort to detect impacts on enzyme functionality, growth on axenic media indicated a delay of the Δ*frd* strain in entering logarithmic phase, a phenotype readily restored to WT levels by complementation with both the BCG Moreau and *M.tb* alleles. Again, no significant differences were found between the complemented strains. FRD and SDH have similarities in structure, mechanisms of action and coenzyme usage, each enzyme prevailing in specific conditions. In *E. coli*, for example, SDH expression is induced during aerobic growth whereas FRD is induced under anaerobic conditions (Cecchini et al., 2002). Furthermore, their nomenclature reflects the reaction direction in which the enzyme displays optimal activity. This activity depends on the strong interaction of each enzyme with the specific quinone, in accordance with its redox potential–ubiquinone and menaquinone are examples of usual quinones interacting with SDH and FRD, respectively (Unden and Bongaerts, 1997).

Mycobacteria are thought to produce only menaquinone and not ubiquinone (Cook et al., 2014), raising the possibility that during evolution the mycobacterial SDH and FRD quinone-interacting cavities evolved to be structurally similar, yielding enzymes with similar activities. Deletion of the *frd* operon could then lead to stress during energy consuming periods, as is the entrance into logarithmic growth phase, since in this period of the bacterial life cycle FRD would be acting as SDH, enhancing the real SDH activity. It is important to emphasize that this would represent a disadvantageous but not necessarily a deleterious event, as the knockout strain would still have SDH/FRD activities provided by *sdh*. *Mycobacterium smegmatis* mc2155 has no *frd* gene but carries two *sdh* homologs to *M.tb* (Pecsi et al., 2014). For *M. smegmatis*, one of the *sdh* alleles is essential, whether for *M.tb* none of them are (DeJesus et al., 2017), maybe due to a compensation mechanism for SDH activity provided by *M.tb frd*.

Looking at the Δ*frd* behavior in an *in vitro* infection context, a discrepancy in growth is still evident, possibly related with its inherent growth delay, as seen in axenic growth, or to a lack of real FRD activity in the oxygen-limiting phagosome environment. The latter hypothesis goes against the results on a *M.tb frdA* knockout obtained by Watanabe et al. (2011), in which there were no significant differences observed for the deleted strain in a mouse model of infection and Wayne model of anaerobiosis assays. According to the authors, this could be explained again as a compensation effect of *sdh1* and *sdh2* operons of *M.tb* on FRD activity in the Δ*frdA* strain. Moreover, *sdh1*, *sdh2*, and *frd* real activities in *M.tb* are still under investigation, as they seem to be functionally redundant under specific conditions, mainly regarding the FRD reaction (Hards et al., 2020). We should keep in mind that the mycobacterial species, experimental models and questions addressed by each study were considerably different, possibly justifying these divergent results–our results are from short-term, BCG *in vitro* responses (less than 1 week), and those reported by Watanabe et al. (2011) are from long-term (30 days) *in vivo M.tb* infections. Furthermore, the Δ*frd* strain was not created in the same way when we compare both set of experiments. For the *M.tb* knockout, only the catalytic subunit gene, *frdA*, was inactivated while we performed a whole operon excision. As speculated, maybe mycobacterial SDH and FRD complexes are more similar than in other bacteria, so catalytic subunits from SDH could be used on FrdA-lacking FRD complexes to restore its activity.

Moreover, the *frd* knockout has a lower internalization rate when compared to the other tested strains, indicating a new role for this complex on the initial steps of the macrophage-bacteria interaction. This also suggests that FRD could behave as a moonlighting protein, performing other relevant physiological activities than the already described (Jeffery, 2018), as other mycobacterial polypeptides such as DnaK (Xolalpa et al., 2007), Hsp65 (Hickey et al., 2009), GroEL1 (Basu et al., 2009) and components of the antigen 85 complex (Belisle, 1997), some of them with extracellular interacting roles. Another possible explanation is that the additional non-canonical FrdB-containing isoforms (FrdBC and FrdBC’D) could perform other functions, participating, for example, on host-bacteria interaction events, corroborating the “new function” scenario of transcriptional slippage advantages on INDEL-affected sequences (Baranov et al., 2005). This could specially be the case with FrdBC’D, carrying a new and specific non-described portion (the out-of-frame *frdC*-coded region), where the whole protein shows increased hydrophobicity, as shown by the differential extraction assays. This new protein could have a novel topology on the bacterial membrane possibly interfering in extracellular events. Further experiments are needed to clarify these questions.

Genes from the *frd* operon have been found to be induced in *M.tb* upon eukaryotic cell internalization. Additionally, the *M.tb* H37Ra attenuated strain shows greater *frd* expression than virulent H37Rv in long term infection (∼168 h p.i.), whether in short-term periods the attenuated strain has lower expression (Li et al., 2008). This corroborates with our data, showing that the *frd* operon may play an important role in these initial infection steps.

Comparing all four strains we could observe that complementation did not lead to identical WT phenotype. During axenic growth, differences were detected on final logarithmic and stationary phases, with complemented strains showing higher absorbance levels. On the other hand, these same strains had a development delay in the macrophage infection model, reaching similar CFU values on the final analyzed time point. Since there are no significant differences between the two complemented strains in both assays, the partial complementation observed should be due to other common factors. Complementation was performed using the integrative vector pMV361H, which carries integration signals from mycobacteriophage L5 (Stover et al., 1991), substituting the vector’s original promoter by the *frd* operon and its own, native promoter. Some studies report that a disadvantage of the L5 integrative vector could be related to the integration locus, which might not favor the transcription of the integrated genes under control of their own, native promoters (Murry et al., 2005; Movahedzadeh et al., 2011). Additionally, the *frd* promoter region included in our complementation constructs may lack other transcriptional regulatory sequences, resulting in a different expression pattern for the *frd* operon. As already known for the *E. coli* homolog (Rabin and Stewart, 1993), anaerobic respiratory enzymes (for example, FRD) are under a hierarchical dual transcriptional regulatory mechanism. A similar multi-step regulation could be acting on mycobacterial FRD expression, for example due to modulation via cAMP responsive protein (CRP) (Bai et al., 2007; Knapp et al., 2015). In consequence, under variable conditions, the wild type BCG strain would modulate FRD expression levels, differing from the complemented strains which would not show these alterations, maintaining the basal FRD levels.

Altogether, our results show that the *frdABCD* operon of Mycobacteria may have evolved to possess other yet non-described functions, both considering the SDH/FRD bifunctionality and the unexpected participation in the early steps of the host-bacteria interaction. Additionally, the evolution of this locus seems to be more prominent in the *M. bovis* lineage, both in the paternal *M. bovis* clinical strain as well as in the attenuated BCG family, a product of *in vitro* evolution. This is possibly due to diminished selective pressure or other neutral evolutionary forces acting on this operon throughout both types of evolutionary history. But independently of the locus analyzed (*M.tb* or BCG), different FrdB-containing polypeptides are expressed that could have resulted from evolutionary forces working on this locus to maintain some ancestral complex function, at the same time producing proteins with new and advantageous characteristics. A better understanding on the dynamics of this locus and the functions of its products could yield very interesting basic knowledge, also applicable to drug discovery, as FRD (specifically the membrane anchors, Rv1554/FrdC and Rv1555/FrdD) is described as a druggable target with predicted interaction with known drugs, such as sildenafil (Viagra), tadalafil (Cialis), and vardenafil (Levitra) (Dash et al., 2018). However, drug design focusing on FRD must take into account inhibition of *sdh1*, *sdh2*, and *frd* products on *M.tb* to overcome their possible function redundancy (Hards et al., 2020). Determining the metabolic role of these different FRD isoforms will help to develop more effective phenotypic assays to detect desirable inhibition effects of tested drugs, making the drug design process faster and more accurate.

## Data Availability Statement

The raw data supporting the conclusions of this article will be made available by the authors, without undue reservation.

## Ethics Statement

The animal study was reviewed and approved by CEUA/IOC, Fiocruz (License L-020/2016).

## Author Contributions

MS, LM-L, WM, CG, EC, and DA conceived and designed the experiments (MS and LM-L: sequence analysis, recombinant protein production and western blot assays; CG, MS, and WM: knockout and complemented strains construction; EC and DA: homology modeling). MS, PC, AS-G, DA, and WM performed the experiments. LM-L, EC, and CG contributed to the reagents, materials, and analysis tools. MS and LM-L wrote the manuscript. All authors analyzed the data, read and approved the final manuscript.

## Conflict of Interest

The authors declare that the research was conducted in the absence of any commercial or financial relationships that could be construed as a potential conflict of interest.

## Footnotes

1 http://servicesn.mbi.ucla.edu/SAVES/

2 https://proteins.plus/

3 https://opm.phar.umich.edu/server

## Supplementary Material

Supplementary Figure 1 Multiple sequence alignment between FRD target proteins and PDB template sequence. Black and gray filled positions in the sequence alignment represent fully and partially conserved residues, respectively. Gaps are represented by points. Chains and GGR link were identified according to the figure legend caption.
