# Supplementary material for: Mycobacterium tuberculosis and M. bovis BCG Moreau Fumarate Reductase Operons Produce Different Polypeptides That May Be Related to Non-canonical Functions
Source: Front Microbiol. 2021 Jan 12;11:624121. doi: 10.3389/fmicb.2020.624121 (PMC7835394; doi:10.3389/fmicb.2020.624121)
Supplement: Supplementary file 1 [file Table_1.DOCX]

**Supplementary Table 1.** BLASTP analysis of the target *M.tb* FRD and selected template structure.

| **PDB ID: 1KF6** | | | | | | |
| --- | --- | --- | --- | --- | --- | --- |
| **Chain** | **Identity** | **Similarity** | **Cover** | **Gaps** | **Max score** | **E-value** |
| A | 55% | 68% | 94% | 1% | 600 | 0.0 |
| B | 50% | 66% | 97% | 0% | 253 | 1e-84 |
| C | 31% | 56% | 98% | 0% | 78.2 | 3e-19 |
| D | 40% | 64% | 60% | 2% | 55.5 | 1e-10 |
