# Supplementary material for: Mycobacterium tuberculosis and M. bovis BCG Moreau Fumarate Reductase Operons Produce Different Polypeptides That May Be Related to Non-canonical Functions
Source: Front Microbiol. 2021 Jan 12;11:624121. doi: 10.3389/fmicb.2020.624121 (PMC7835394; doi:10.3389/fmicb.2020.624121)
Supplement: Supplementary file 2 [file Table_2.DOCX]

Supplementary Table 2. Evaluation of FRD models.

| **Models** | **QMean** | **ERRAT2** | | | | **Ramachandran Plot** | | | |
| --- | --- | --- | --- | --- | --- | --- | --- | --- | --- |
|  |  | **A** | **B** | **C** | **D** | **R1** | **R2** | **R3** | **R4** |
| **1KF6** | -1.57 | 95.9578 | 93.5857 | 91.1504 | 100 | 88.0 | 11.4 | 0.5 | 0.1 |
| **Frd_MTB** | -2.84 | 76.3066 | 65.6904 | 75 | 88.8889 | 92.0 | 6.6 | 0.9 | 0.5 |
| **Frd_BCG** | -3.33 | 77.5652 | 63.5359 | | 91.453 | 91.1 | 7.4 | 1.1 | 0.4 |

R1: Residues in most favored regions; R2: Residues in additional allowed regions; R3: Residues in generously allowed regions; R4: Residues in disallowed regions.
