# Supplementary figures and images for: Mycobacterium tuberculosis and M. bovis BCG Moreau Fumarate Reductase Operons Produce Different Polypeptides That May Be Related to Non-canonical Functions
Source: Front Microbiol. 2021 Jan 12;11:624121. doi: 10.3389/fmicb.2020.624121 (PMC7835394; doi:10.3389/fmicb.2020.624121)

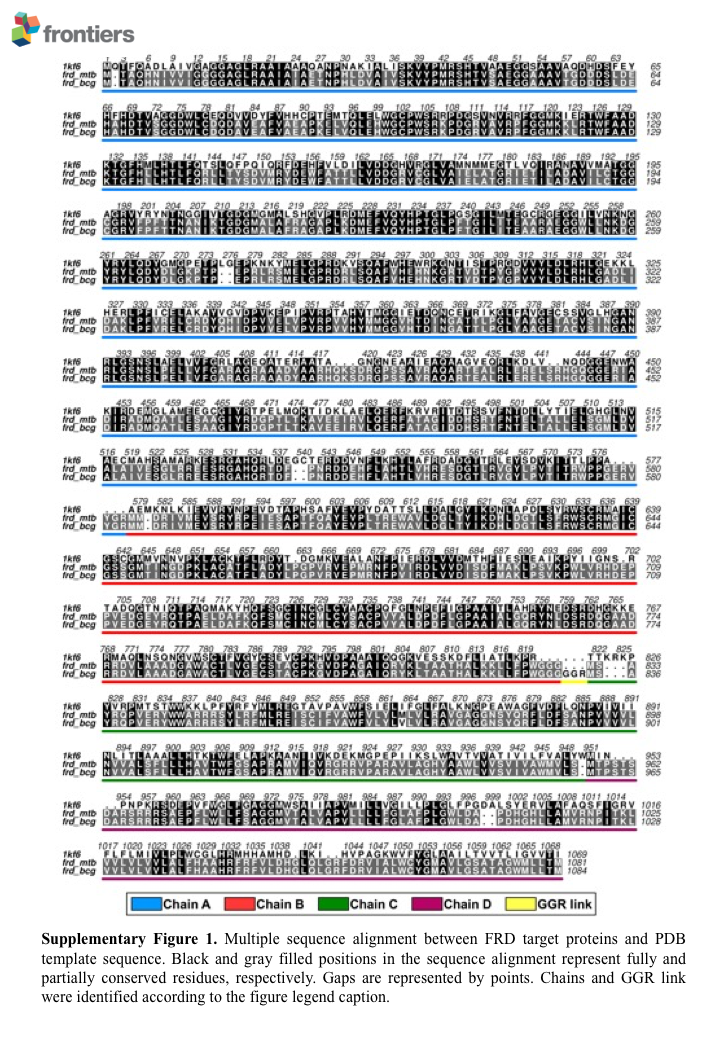

Supplement: Supplementary Figure 1 — Multiple sequence alignment between FRD target proteins and PDB template sequence. Black and gray filled positions in the sequence alignment represent fully and partially conserved residues, respectively. Gaps are represented by points. Chains and GGR link were identified according to the figure legend caption. [file Image_1.tiff]
